# Association of hypertensive disorders of pregnancy with offspring cardiometabolic indicators: a systematic review and meta-analysis

**DOI:** 10.3389/fendo.2025.1641563

**Published:** 2025-11-03

**Authors:** Hong Xu, Yujie Liang, Peishan Li

**Affiliations:** Department of Obstetrics and Gynecology, Chengdu Shuangliu District Maternal and Child Health Hospital, Chengdu, China

**Keywords:** hypertensive disorders of pregnancy, HDP, cardiometabolic, offspring, meta-analysis

## Abstract

**Background:**

Hypertensive disorders of pregnancy (HDP) are a major public health problem affecting a large number of pregnancies worldwide. Despite extensive research, little is known about the long-term cardiometabolic consequences of HDP exposure in offspring.

**Objective:**

To investigate the long-term cardiometabolic risks in offspring exposed to HDP.

**Search strategy:**

A comprehensive search of relevant studies published in PubMed, EMBASE, the Cochrane Central Register, and Web of Science databases was conducted.

**Selection criteria:**

Inclusion criteria comprised case-control and cohort studies, with outcome measures encompassing blood pressure, body mass index, lipid levels, and glucose metabolism.

**Data collection and analysis:**

Meta-analysis was performed using Review Manager 5.4, and fixed- or random-effects models were selected as appropriate.

**Main results:**

A total of 23 observational studies with 89,982 participants from 10 countries were included. Meta-analysis indicated that offspring exposed to HDP presented with significantly increased systolic blood pressure (MD: 2.44; 95% CI: 2.03–2.85; *P* < 0.00001), elevated diastolic blood pressure (SMD: 0.19; 95% CI: 0.15–0.23; *P* < 0.00001), and higher body mass index (MD: 0.34; 95% CI: 0.05–0.64; *P* < 0.05). Additionally, these offspring demonstrated a decreased likelihood of elevated homeostasis model assessment of insulin resistance (OR: 0.58; 95% CI: 0.34–0.98; *P* < 0.05). No significant differences were observed in other indicators.

**Conclusions:**

The impact of HDP on offspring cardiometabolism is multifaceted. Elevated blood pressure and body mass index are more likely to be observed in offspring exposed to HDP, while the risk of insulin resistance appears to be reduced.

**Systematic review registration:**

https://www.crd.york.ac.uk/PROSPERO/view/CRD42025630378, identifier CRD42025630378.

## 1 Introduction

Hypertensive disorders of pregnancy (HDP) encompass a range of conditions characterized by elevated blood pressure during pregnancy, affecting 5%–10% of pregnancies globally (1, 2). These include pregnancy-induced hypertension, preeclampsia, eclampsia, and hemolysis, elevated liver enzymes, and low platelets (HELLP) syndrome (3). In the United States, the occurrence of HDP increased from 13% in 2017 to 16% in 2019. During this period, preeclampsia complications were identified in approximately 3% of all pregnancies. There was a notable rise in HDP cases between 2017 and 2019 (4). Globally, these complications occur in 2%–8% of pregnancies (5). HDP poses immediate health risks to pregnant women, such as higher cesarean section rates, placental abruption, increased maternal mortality, and long-term cardiovascular disease, and significantly impacts fetal development, leading to intrauterine growth restriction, preterm birth, and elevated perinatal mortality (6–12).

The impact of HDP on children’s cardiometabolic health remains a subject of debate. There are differing viewpoints regarding the long-term consequences of HDP exposure for offspring. Some studies report significant associations between HDP and elevated blood pressure among offspring. For instance, a comprehensive systematic review conducted by Davis et al. (13) indicated that children exposed to preeclampsia exhibited increased systolic and diastolic blood pressure during childhood and young adulthood. Similarly, a prospective study by Alsnes et al. (14) found that such exposure was linked to higher blood pressure levels in offspring. However, other studies, such as those by Jansen et al. (15) and Tripathi et al. (16), found no significant or only weak associations after adjusting for maternal characteristics. Findings on metabolic indicators are also inconsistent. While Alsnes et al. (14) reported higher body mass index (BMI) in HDP-exposed offspring during adolescence, Tripathi et al. (16) reached the opposite conclusion, and no consensus exists regarding the relationship between HDP and other metabolic indicators, such as blood lipids and glucose (16, 17).

Given the diverse findings and their public health implications, systematic reviews and meta-analyses are essential to synthesize current evidence and accurately assess the effects of HDP on offspring cardiometabolic health. This approach clarifies the strength of the association between HDP and offspring cardiovascular health, provides a foundation for early preventive interventions, and informs the monitoring and management of long-term health outcomes in affected offspring. Understanding these associations is particularly important given the rising incidence of hypertensive disorders in pregnancy and the global burden of cardiovascular and metabolic diseases.

## 2 Methods

This meta-analysis strictly adhered to the PRISMA (Preferred Reporting Items for Systematic Reviews and Meta-Analyses) and MOOSE (Meta-analysis of Observational Studies in Epidemiology) guidelines. The review protocol was registered with PROSPERO on January 17, 2025 (registration number: CRD42025630378).

### 2.1 Literature retrieval

The literature search included online searches of published databases and archives, with authors contacted when necessary. This review focused on the relationship between hypertensive conditions during pregnancy and cardiometabolic outcomes in offspring, specifically examining population-based links between these hypertensive disorders and their potential impact on offspring cardiometabolic health.

Four comprehensive electronic databases—PubMed, EMBASE, the Cochrane Central Register of Controlled Trials, and Web of Science—were searched from their inception through December 2024. The search strategy combined subject headings and free-text terms. Keywords included *hypertension*, *pregnancy-induced hypertension*, *cardiovascular system*, *cardiovascular diseases*, BMI, *blood pressure*, *triglycerides*, *cholesterol*, and offspring.

The retrieval strategy, using PubMed as an example, was as follows: #1: Hypertension, Pregnancy-Induced[MeSH Terms],#2: (((((((((((Hypertension, Pregnancy-Induced[Title/Abstract]) OR (Hypertension, Pregnancy Induced[Title/Abstract])) OR (Pregnancy-Induced Hypertension[Title/Abstract])) OR (Gestational Hypertension[Title/Abstract])) OR (Hypertension, Gestational[Title/Abstract])) OR (Pregnancy Induced Hypertension[Title/Abstract])) OR (Induced Hypertension, Pregnancy[Title/Abstract])) OR (Transient Hypertension, Pregnancy[Title/Abstract])) OR (Hypertension, Pregnancy Transient[Title/Abstract])) OR (Pregnancy Transient Hypertension[Title/Abstract])) OR (HDP[Title/Abstract])) OR (HDPS[Title/Abstract]),#3: #1 OR #2,#4: ((((Cardiovascular System[MeSH Terms]) OR (Cardiovascular Diseases[MeSH Terms])) OR (Cardiometabolic Risk Factors[MeSH Terms])) OR (Metabolic Syndrome[MeSH Terms])) OR (Endocrine System[MeSH Terms]),#5: ((((((((((((((((((Cardiovascular System[Title/Abstract]) OR (Cardiovascular Diseases[Title/Abstract])) OR (Cardiometabolic Risk Factors[Title/Abstract])) OR (Metabolic Syndrome[Title/Abstract])) OR (Endocrine System[Title/Abstract])) OR (Syndromes, Metabolic[Title/Abstract])) OR (cardiovascular health[Title/Abstract])) OR (Cardiovascular diseases[Title/Abstract])) OR (Disease, Cardiovascular[Title/Abstract])) OR (metabolic health[Title/Abstract])) OR (Glucose[Title/Abstract])) OR (Birthweight[Title/Abstract])) OR (BMI[Title/Abstract])) OR (Insulin[Title/Abstract])) OR (fasting insulin[Title/Abstract])) OR (Insulin Resistance[Title/Abstract])) OR (Cholesterol[Title/Abstract])) OR (Triglycerides[Title/Abstract])) OR (Blood Pressure[Title/Abstract]),#6: #4 OR #5,#7: Child[MeSH Terms],#8: (((Child[Title/Abstract]) OR (Children[Title/Abstract])) OR (Childhood[Title/Abstract])) OR (Offspring[Title/Abstract]),#9: #7 OR #8,#10: #3 AND #6 AND #9.

### 2.2 Inclusion criteria

(1) Case-control or cohort studies;
(2) Content: Relationship between hypertensive disorders in pregnancy and offspring cardiometabolic outcomes;
(3) Population: Pregnant women with hypertensive disorders;
(4) Outcomes: At least one of the following—systolic blood pressure (SBP), diastolic blood pressure (DBP), body mass index (BMI), waist circumference, fat mass index, triglycerides, high-density lipoprotein (HDL), low-density lipoprotein (LDL), total cholesterol, blood glucose, or homeostasis model assessment of insulin resistance (HOMA-IR).

### 2.3 Exclusion criteria

(1) Non–case-control and non-cohort studies (e.g., animal studies, protocols, abstracts, case reports, correspondence);
(2) Studies not meeting diagnostic criteria for hypertensive disorders in pregnancy;
(3) Incomplete data with unextractable odds ratios and 95% confidence intervals (CIs);
(4) Duplicate publications;
(5) Unavailable full text.

### 2.4 Literature screening

Bibliographic management software (EndNote) was used to organize document selection and exclusion. Abstracts and full texts were independently reviewed by two researchers (YL and PL) to determine eligibility, and full texts of potentially eligible studies were retrieved and assessed. Two researchers (YL and PL) independently read the full texts of all potentially eligible articles and identified those meeting the inclusion criteria to ensure accuracy. In case of any discrepancies in inclusion decisions, consensus was reached through discussion or with the assistance of a third independent researcher (HX).

### 2.5 Data extraction

After reviewing the full texts of studies meeting the inclusion criteria, data were independently extracted by two researchers (YL and PL). Extracted data on the cardiometabolic effects of HDP on offspring included study name (first author and publication year), country or region, study design, study population, follow-up period, exclusion criteria, outcome measures, adjustment factors, and other relevant information.

### 2.6 Literature quality evaluation

The quality of included studies was independently assessed by two researchers (HX and YL) using the Newcastle–Ottawa Scale (NOS). The scale includes three categories—population selection, comparability, and exposure or outcome evaluation—with a maximum score of 9 points. The scoring system was classified as follows: scores of ≤3 indicated low quality, scores of 4–6 indicated medium quality, and scores of ≥7 reflected high quality. Discrepancies were resolved through discussion or by consulting a third investigator (PL).

### 2.7 Statistical methods

Meta-analysis was conducted using Review Manager 5.4. For categorical variables, odds ratios (ORs) with 95% confidence intervals (CIs) were used. Continuous variables were evaluated using mean difference (MD) or, as needed, standardized mean difference (SMD), each accompanied by 95% CIs. MD was used for all analyses, while SMD was applied when combining data from studies using different scales. A fixed-effects model was employed when *I*² < 50%; when *I*² ≥ 50%, a random-effects model was adopted (18). Publication bias was assessed using funnel plots.

## 3 Results

### 3.1 Overview of search results

**Figure 1:**
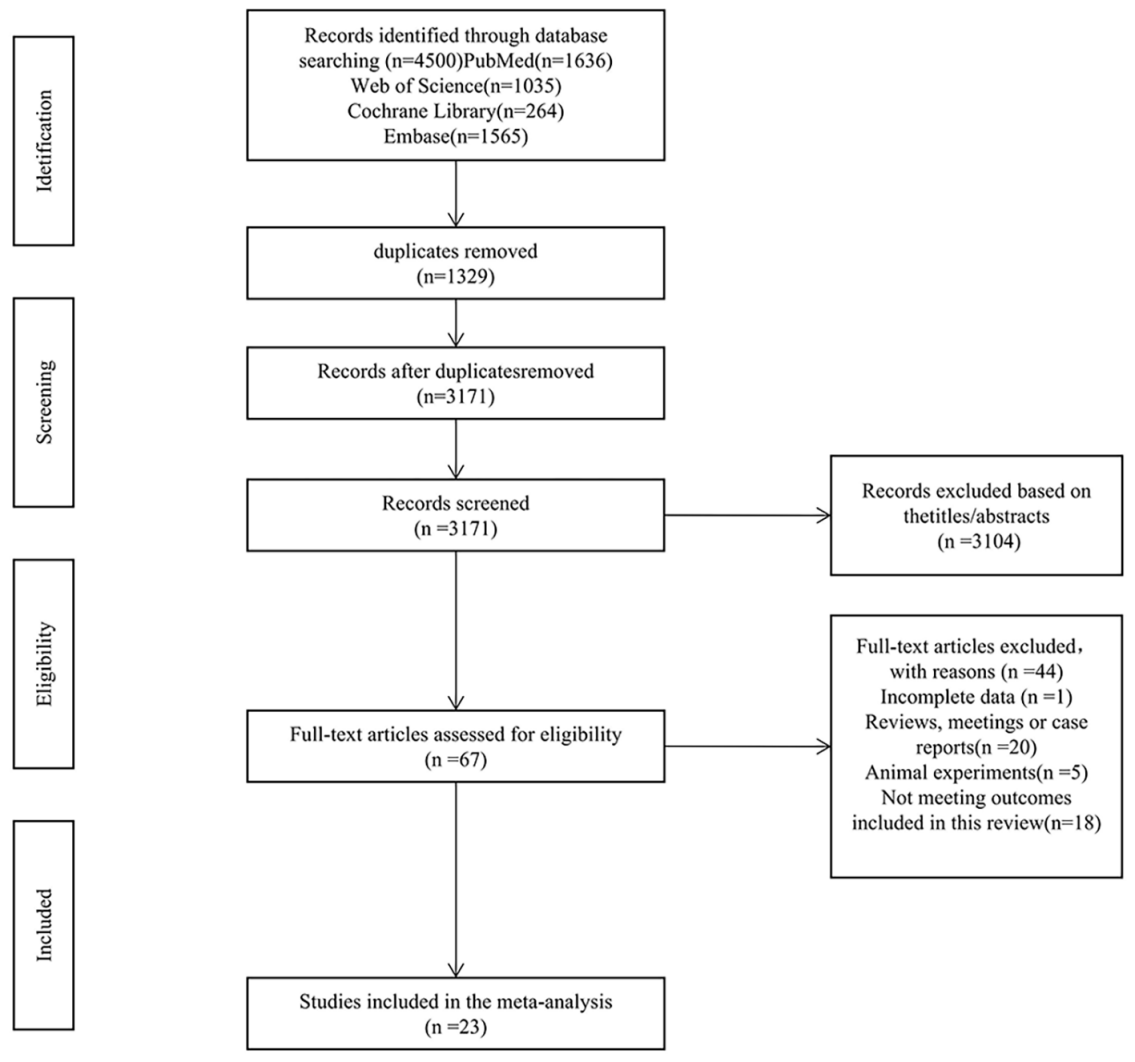
PRISMA flow diagram of included and excluded studies.

The initial search yielded 4,500 records. After eliminating 1,329 duplicates and dismissing 3,104 irrelevant records according to titles and abstracts, 67 full-text articles were evaluated. Among these, 44 were excluded: incomplete data (n = 1), reviews (n = 11), conference abstracts (n = 5), case reports (n = 4), animal studies (n = 5), and failure to meet outcome indicators (n = 18). Ultimately, 23 studies satisfied the eligibility requirements. The selection procedure is presented in **Figure 1**.

### 3.2 Overview of included studies

A total of 23 observational studies (16, 17, 19–39) were included, comprising 21 prospective cohort studies, 1 retrospective cohort study, and 1 nested case-control study. These studies involved 89,982 participants (10,854 with gestational hypertension and 79,128 with normal blood pressure) from 10 countries: 4 studies in China, 4 in Finland, 4 in the Netherlands, 3 in the United Kingdom, 2 in the United States, 2 in Australia, and 1 each in Southern Denmark, Japan, Norway, and Lithuania. Of the included studies, 18 reported systolic blood pressure (SBP), 17 reported diastolic blood pressure (DBP), 14 reported body mass index (BMI), 6 reported waist circumference, 5 reported blood glucose, 6 reported high-density lipoprotein (HDL), 2 reported low-density lipoprotein (LDL), 4 reported triglycerides, and 5 reported total cholesterol. Two studies assessed offspring fat mass index, and two studies evaluated offspring HOMA-IR. The detailed characteristics of the 23 included studies are presented in **Table 1**.

**Table 1:** Summary of the studies included in the meta-analysis.

Author(year)(ID)	Country/Region	Study design	Population	Follow-up Period Age(year) mean(SD)/median(IQR)	Exclusion criteria	Outcomes	Adjusted Factors
Ruby Reetika Tripathi (2018)(16)	Massachusetts, USA	Prospective cohort study	Normal: 98 PE or GH: 964 Recruitment period: 1999-2002	Normal: 7.9 (0.9) PE or GH: 8.1 (0.9)	Multiple Pregnancies,Non-English Speaking Ability,Planning to Move Away During Follow-up,First Prenatal Visit After 22 Weeks,mothers with diabetes,use antihypertensive medications during preg- Nancy,without any information on hypertensive disorders of pregnancy or data on prenatal blood pressures	SBP DBP LDL HOMA-IR fat mass index	child age,sex,birth weight for gestational age z-score,maternal race/ethnicity, age at enrollment, height and prepregnancy BMI, education, household income, parity and smoking during pregnancy
Madeline Murguia Rice (2018)(19)	USA	Prospective observational follow-up study	Normotensive: 73 Pregnancy-Associated Hypertension: 825 Recruitment period: 2012-2013	Normotensive:7 (6–8) Pregnancy-Associated Hypertension: 7 (6–8)	NA	SBP DBP HDL Triglyceride Glucose HOMA-IR BMI Waist circumference	NA
Robyn J. Tapp (2018)(20)	Finland	Prospective cohort study	Without HDP: 877 HDP: 129 Recruitment period: 2011-2012	Without HDP: 41.6 (5.0) HDP: 39.7 (4.6)	Premature infants born before 37 weeks of gestation,Infants not appropriate for gestational age (birth weight not between 50th-90th percentile),Participants who did not complete the following examinations(Retinal photography,Cardiac assessment),Participants lacking the data	SBP DBP HDL Total cholesterol, Glucose HOMA-IR BMI Waist circumference	age, sex, birth weight, BMI, smoking, SES, and hypertension
S Zhang (2017)(21)	Tianjin,China	Prospective cohort study	Without HDP: 1172 HDP: 91 Recruitment period: 2009-2011	Age (months): Without HDP: 26.8 (10.5) HDP: 28.1 (10.3)	NA	BMI	NA
Abigail Fraser (2013)(22)	United Kingdom	Prospective cohort study	Normotensive: 2404 Hypertension:431 Preeclampsia:53 Recruitment period: 2006-2009	Age (months): Normotensive: 208.5 (11.1) Hypertension:208.1 (11.7) Preeclampsia:209.9 (10.9)	NA	SBP DBP HDL LDL Total cholesterol Glucose BMI	NA
Satu Miettola (2013)(23)	Finland	Prospective cohort study	Normotensive: 5045 Hypertension:331 Preeclampsia:197 Recruitment period: 2001-2002	Normotensive: 16 Hypertension:16 Preeclampsia:16	Subjects on antihypertensive medication at the beginning of pregnancy were excluded, as were subjects with BP C 140/90 before week 20 because they were considered to have chronic hyperten- sion. A positive urinary dip-stick test (C0.3 g/L) indicated proteinuria. Normotensive subjects (BP\140/90) with proteinuria were excluded from the study	SBP DBP HDL LDL Triglyceride Total cholesterol Glucose BMI Waist circumference	NA
Clarissa J. Wiertsema (2022)(24)	Rotterdam, The Netherlands	Prospective cohort study	Normotensive: 4410 Hypertension:184 Preeclampsia:85 Recruitment period: 2001-2002	Age(year) median(95%range) Normotensive: 9.7(9.4 to 10.7) Hypertension:9.7(9.3 to 11.2) Preeclampsia:9.7(9.4 to 10.8)	Women with pre-existing hypertension,Multiple pregnancies,Non-singleton pregnancies,Non-live births,Offspring with cardiac abnormalities,Cases with missing exposure data,Cases without follow-up at age 10 years Children without any cardiovascular measurements at follow-up	SBP DBP	NA
Anna Birukov (2020)(25)	Odense, southern Denmark	Prospective cohort study	Normal: 1512 GH: 66 Recruitment period: 2010-2012	Normal: 5 GH: 5	NA	SBP DBP HDL LDL	maternal prepregnancy BMI, maternal age, smoking status, parity, log-transformed angiogenic marker concentrations at gestational age 28, offspring BMI, birth weight
Christina Y. L. Aye (2020)(26)	United Kingdom	Prospective cohort study	Normotensive: 54 Hypertension:80 Recruitment period: 2011-2015	Age(day): Normotensive: 99.6±14.8 Hypertension:96.7±13.1	Maternal Exclusion Criteria:Age below 16 years old,History of chronic cardiovascular diseases,Essential hypertension Infant Exclusion Criteria:Severe malformations,Congenital cardiovascular disease,Chromosomal abnormalities,Genetic disorders	SBP DBP	NA
Benjamin J. Varley (2022)(27)	Australia	Cross-sectional study (sub-study of a prospective cohort study)	Normotensive: 34 Preeclampsia:26 Recruitment period: 2020-2021	Normotensive:5.0 (2.2, 5.3) Preeclampsia:4.3 (3.2, 5.1)	Women were excluded if they had chronic hypertension, diabetes, renal or other serious disease prior to pregnancy, were pregnant again at the time of first (6 months postpartum) assessment, or if their baby was born with a congenital anomaly	BMI	NA
Kritika Poudel (2021)(28)	Japan	Prospective cohort study	Without HDP: 6085 HDP: 118 Recruitment period: 2003-2012	Without HDP: 7.01(0.32) HDP: 7.01(0.25)	Mothers with miscarriages, stillbirths, abortions, twins, and multiple births,mothers who did not have information on parity and anthropometry of their children at birth were excluded; Children with no information on anthropometric measurements at one, two, four, and seven years were excluded	BMI	NA
Yuying Gu (2019)(29)	China	Prospective cohort study	Without HDP: 677 HDP: 27 Recruitment period: 2010-2013	Without HDP: 5.25(1.20) HDP: 5.41(1.31)	NA	BMI	NA
Dionne V. Gootjes (2021)(30)	Rotterdam, The Netherlands	Prospective cohort study	Without HDP: 7303 HDP: 491 Recruitment period:2002-2006	Without HDP: 6 HDP: 6	twin pregnancies, terminated pregnancies, intra-uterine fetal demise and pregnancies without data on maternal hypertensive disorders or early childhood cardiometabolic risk factors	SBP DBP Triglyceride Total cholesterol BMI	child's sex,maternal pre-pregnancy body mass index, educational level, ethnicity, smoking during pregnancy, alcohol use during pregnancy, maternal glucose levels and presence of gestational diabetes mellitus.
Kristine Kjer Byberg (2017)(31)	Norway	Follow-up of a nested case-control study	Normotensive: 385 Preeclampsia:229 Recruitment period:1993-1995	Normotensive/ Preeclampsia:First follow-up at the ages of 10.8 years (girls) and 11.8 years (boys); Second follow-up at 12.8 years	Cases of twin or multiple pregnancies,Children with chronic diseases,Children with chromosomal abnormalities,Children with congenital malformations,Cases with incomplete growth and development records,Cases with missing key clinical information,Cases with unclear preeclampsia diagnosis,Cases that did not meet the diagnostic criteria for preeclampsia	BMI	Child's sex,Birth order,firstborn or not,Maternal age at delivery,Maternal BMI,Maternal smoking in pregnancy,Maternal education at delivery
Meddy N. Bongers-­Karmaoui (2023)(32)	the Netherlands	Prospective cohort study	Normotensive: 2322 GH:87 Recruitment period:2001-2006	Normotensive: 9.9 (9.8-10.3) GH:10.0 (9.8-11.0)	pre-existent hypertension,non-singleton pregnancies,non-live births,cardiac anomaly,Children who failed to complete cardiac magnetic resonance imaging,Children with suboptimal quality of cardiac magnetic resonance imaging	SBP DBP	NA
Michelle A.-K. Renlund (2023)(33)	Finland	Prospective cohort study	non-PE:85 PE:182 Recruitment period:2019-2022	non-PE:11.2 (1.0) PE:11.6 (1.1)	Ongoing maternal pregnancy or lactation, Multiple pregnancy, Inability to communicate in Finnish	SBP	NA
Fengxiu Ouyang (2023)(34)	Shanghai, China	Prospective cohort study	Normal: 402 GH: 26 Recruitment period:2014-2015	Normal: 2 GH: 2	Multiple pregnancy, Not having routine prenatal care at study hospitals, Planning to leave Shanghai in next 2 years, Unwilling to participate or provide informed consent, Preterm births, gestational age < 37 weeks, Children without follow-up data	SBP DBP HDL LDL Triglyceride Total cholesterol Glucose	child age, sex, and weight-for-length z-score
Yan Chen (2023)(35)	China	Prospective cohort study	Normotension: 30309 Mild GH:3180 Recruitment period:1959-1976	Normotension: 7 Mild GH: 7	Non-live births, Multiple pregnancies, missing blood pressure records and diagnostic information, missing birth weight data, missing key covariate data, missing 7-year follow-up data	SBP DBP BMI	parity, diabetes, maternal education level, race, sex, marital status, smoking status during pregnancy, socioeconomic status, maternal pre-pregnancy body mass index (BMI), gestational weight gain, and gestational age, birth weight and BMI at 7 years of age
Renata Kuciene (2022)(36)	Kaunas City, Lithuania	Retrospective cohort study	Normotensive: 4448 GH:371 Recruitment period:2010-2012	Normotensive: 13.61(1.04) GH:13.55(1.06)	endocrine diseases, kidney diseases, congenital heart defects, cardiovascular diseases, missing anthropometric measurements, missing data on birth weight and gestational age, multiple births	SBP DBP	age, sex, birth weight, adolescent BMI, and maternal pre/early pregnancy BMI
Debbie Anne Lawlor (2011)(37)	UK	Prospective cohort study	No HDP: 5367 HDP:1182 Recruitment period:1991-1992	Age (months): No HDP:118 (4) HDP:118 (4)	Non-singleton Pregnancy,Neonates who died within one year after birth,Maternal-infant pairs lost to follow-up	SBP DBP HDL BMI Waist circumference	NA
Esther F Davis (2015)(38)	Australia	Prospective birth cohort study	Normotensive: 899 Hypertensive:252 Recruitment period:1989-1991	Normotensive: 20 Hypertensive:220	Congenital anomaly,Mothers with a history of hypertension before pregnancy,Data Missing	SBP DBP	birth weight, gestational Age, infant sex, mode of delivery, maternal age, maternal BMI and maternal smoking in late pregnancy,risk factors at age 20 (BMI, cholesterol, insulin and smoking status),ssocioeconomic status at 20 years of age, average weekly alcohol consumption and contraceptive pill use
J.J. Miranda Geelhoed (2010)(17)	the Netherlands	Prospective birth cohort study	Normotensiv: 5082 GH:1065 Recruitment period:1991-1992	Age (months): Normotensiv: 118.0 (114.0–128.0) GH:118.5 (114.0–129.0)	Multiple pregnancy,Stillbirth or neonatal death cases,Cases without permission to extract obstetric record data,Cases lost to follow-up or not completed at the age of 9 years,Cases with incomplete key data	SBP DBP BMI Waist circumference	offspring sex and age at the 9-year visit,maternal age at delivery, parental prepregnancy BMI, parity, social class, and maternal smoking during pregnancy, plus offspring weight, height, and height squared at the 9-year visit,mode of delivery, gestational age at birth, and birth weight
Michelle Renlund-­Vikström (2024)(39)	Helsinki, Finland	Prospective cohort study	Non-PE: 85 PE:182 Recruitment period:2019-2022	Non-PE: 11.2(1.0) PE:11.6(1.1)	mothers’ inability to communicate in Finnish,ongoing pregnancy or lactation, and multiple pregnancy. Exclusion criteria for mothers without preeclampsia were additionally preeclampsia,gestational hypertension, chronic hypertension, gestational diabe- tes or diabetes during or after the index pregnancy	SBP DBP BMI Waist circumference	NA

### 3.3 Methodological quality of included studies

Risk of bias was assessed for the included studies. The 23 articles were evaluated according to the three domains of the Newcastle–Ottawa Scale (NOS) (40). All 23 studies included clearly defined and representative patients with hypertension during pregnancy, a control group of pregnant women with normal blood pressure, and reasonable settings with clear definitions. All 23 studies were comparable, with 10 adjusting key parameters. One study was retrospective with inconclusive exposure assessment, while the remaining 22 studies were prospective. In one study, the follow-up loss rate was slightly higher due to use of questionnaires. Overall, all 23 studies scored above 7 points, indicating high quality (**Table 2**).

**Table 2:** Literature quality evaluation.

Study,first author,year	Selection				Exposure			Non-response rate	Score
	Is the case definition adequate?	Representativeness of the cases	Selection of controls	Definition of controls	Comparability Control for important factor	Ascertainment of exposure	Same method of ascertainment for cases and controls		
Ruby Reetika Tripathi (2018)	★	★	★	★	★★	★	★	★	9
Madeline Murguia Rice (2018)	★	★	★	★	★	★	★	★	8
Robyn J. Tapp (2018)	★	★	★	★	★	★	★	★	8
S Zhang (2017)	★	★	★	★	★	★	★	★	8
Abigail Fraser (2013)	★	★	★	★	★	★	★	★	8
Satu Miettola (2013)	★	★	★	★	★	★	★	★	8
Clarissa J. Wiertsema (2022)	★	★	★	★	★	★	★		7
Anna Birukov (2020)	★	★	★	★	★★	★	★	★	9
Christina Y. L. Aye (2020)	★	★	★	★	★	★	★	★	8
Benjamin J. Varley (2022)	★	★	★	★	★	★	★	★	7
Kritika Poudel (2021)	★	★	★	★	★	★	★	★	8
Yuying Gu (2019)	★	★	★	★	★	★	★	★	8
Dionne V. Gootjes (2021)	★	★	★	★	★★	★	★	★	9
Kristine Kjer Byberg (2017)	★	★	★	★	★★	★	★	★	9
Meddy N. Bongers-Karmaoui (2023)	★	★	★	★	★★	★	★	★	9
Michelle A.-K. Renlund (2023)	★	★	★	★	★★	★	★	★	9
Fengxiu Ouyang (2023)	★	★	★	★	★★	★	★	★	9
Yan Chen (2023)	★	★	★	★	★★	★	★	★	9
Renata Kuciene (2022)	★	★	★	★	★★		★	★	8
Debbie Anne Lawlor (2011)	★	★	★	★	★	★	★	★	8
Esther F Davis (2015)	★	★	★	★	★★	★	★	★	9
J.J. Miranda Geelhoed (2010)	★	★	★	★	★	★	★	★	8
Michelle Renlund-Vikström (2024)	★	★	★	★	★	★	★	★	8

### 3.4 Statistical results

#### 3.4.1 Results: impact of HDP on offspring blood pressure

**Figure 2:**
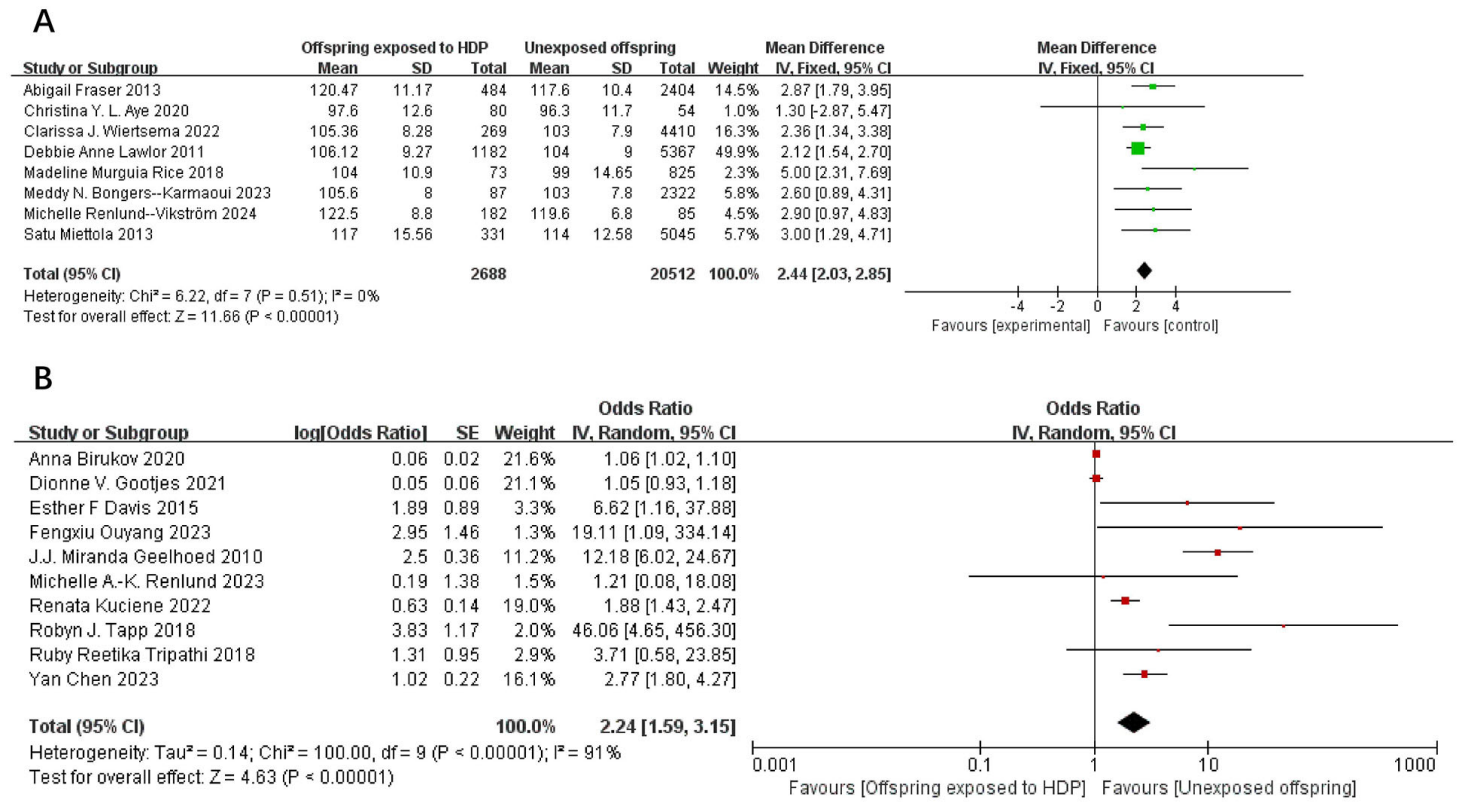
The forest plot shows the impact of HDP on offspring SBP **(A)** continuous variable **(B)** categorical variable.

A total of 75,980 participants from 18 articles were included to assess the influence of HDP on offspring SBP. A higher offspring SBP is associated with a greater risk. As shown in **Figures 2A, B**, in 8 studies (19, 22–24, 26, 32, 37, 39), *I*² = 0% prompted a fixed-effect model, yielding an MD of 2.44 (95% CI: 2.03–2.85; *P* < 0.00001). In 10 studies (16, 17, 20, 25, 30, 33–36, 38), *I*² = 91% led to a random-effects model, yielding an OR of 2.24 (95% CI: 1.59–3.15; *P* < 0.00001). Both results were statistically significant.

**Figure 3:**
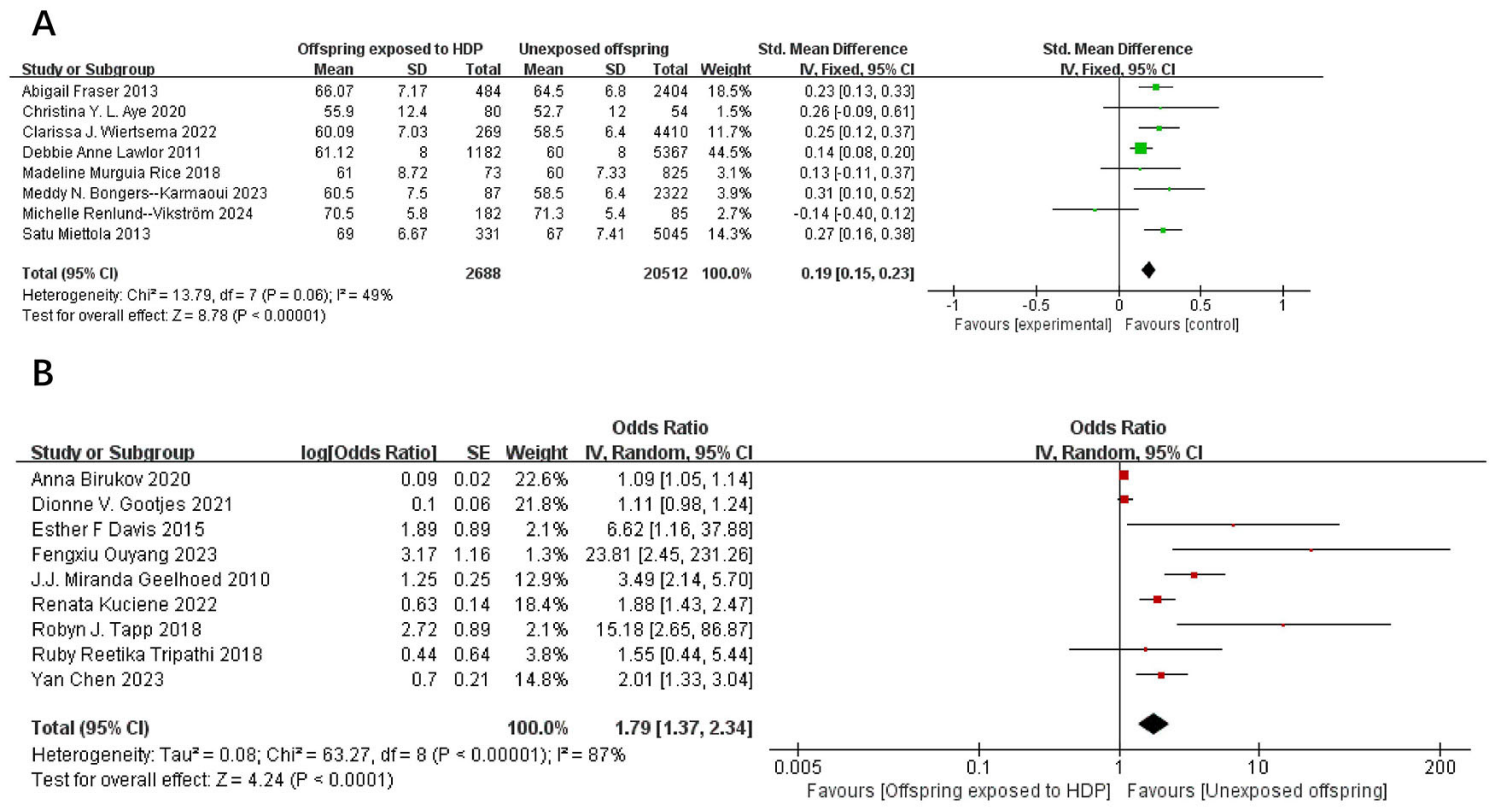
The forest plot shows the impact of HDP on offspring DBP, **(A)** continuous variable **(B)** categorical variable.

A total of 75,713 participants from 17 articles were included to assess the influence of HDP on offspring DBP. Higher offspring DBP is associated with greater risk. As shown in **Figure 3A, B**, in 8 studies (19, 22–24, 26, 32, 37, 39), *I*² = 49% prompted a fixed-effect model, yielding an SMD of 0.19 (95% CI: 0.15–0.23; *P* < 0.00001). In nine studies (16, 17, 20, 25, 30, 34–36, 38), *I*² = 87% led to a random-effects model, yielding an OR of 1.79 (95% CI: 1.37–2.34; *P* < 0.00001). Both results were statistically significant.

#### 3.4.2 Results: impact of HDP on offspring obesity

**Figure 4:**
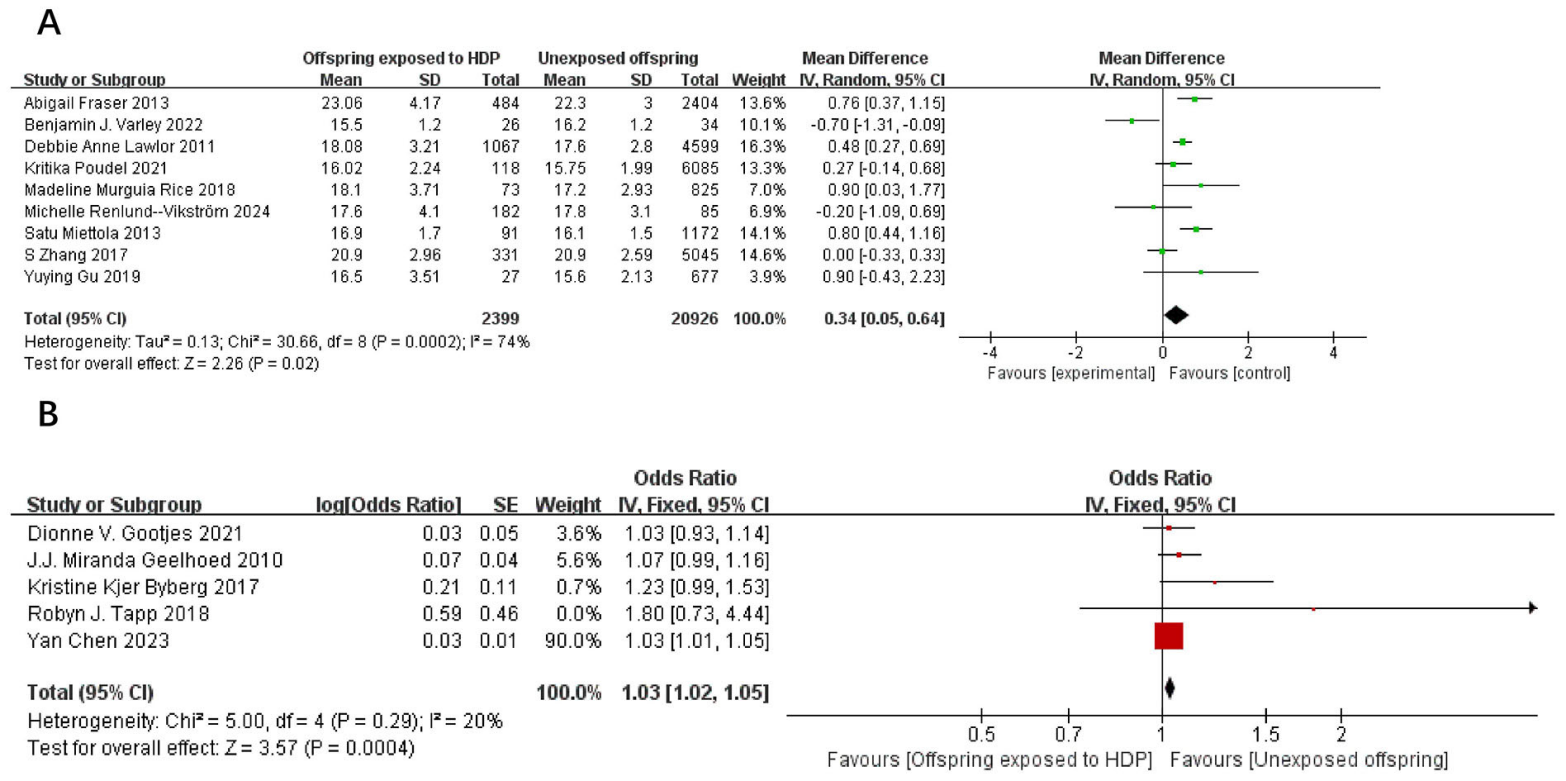
The forest plot shows the impact of HDP on offspring BMI, **(A)** continuous variable **(B)** categorical variable.

A total of 67,180 participants from 14 articles were included to assess the influence of HDP on offspring BMI. Higher offspring BMI is associated with greater risk. As shown in **Figure 4A, B**, in 9 studies (19, 21–23, 27–29, 37, 39), *I*² = 74% prompted a random-effects model, yielding an MD of 0.34 (95% CI: 0.05–0.64; *P* = 0.02). In five studies (17, 20, 30, 31, 35), *I*² = 20% prompted a fixed-effect model, yielding an OR of 1.03 (95% CI: 1.02–1.05; *P* = 0.0004). Both results were statistically significant.

**Figure 5:**
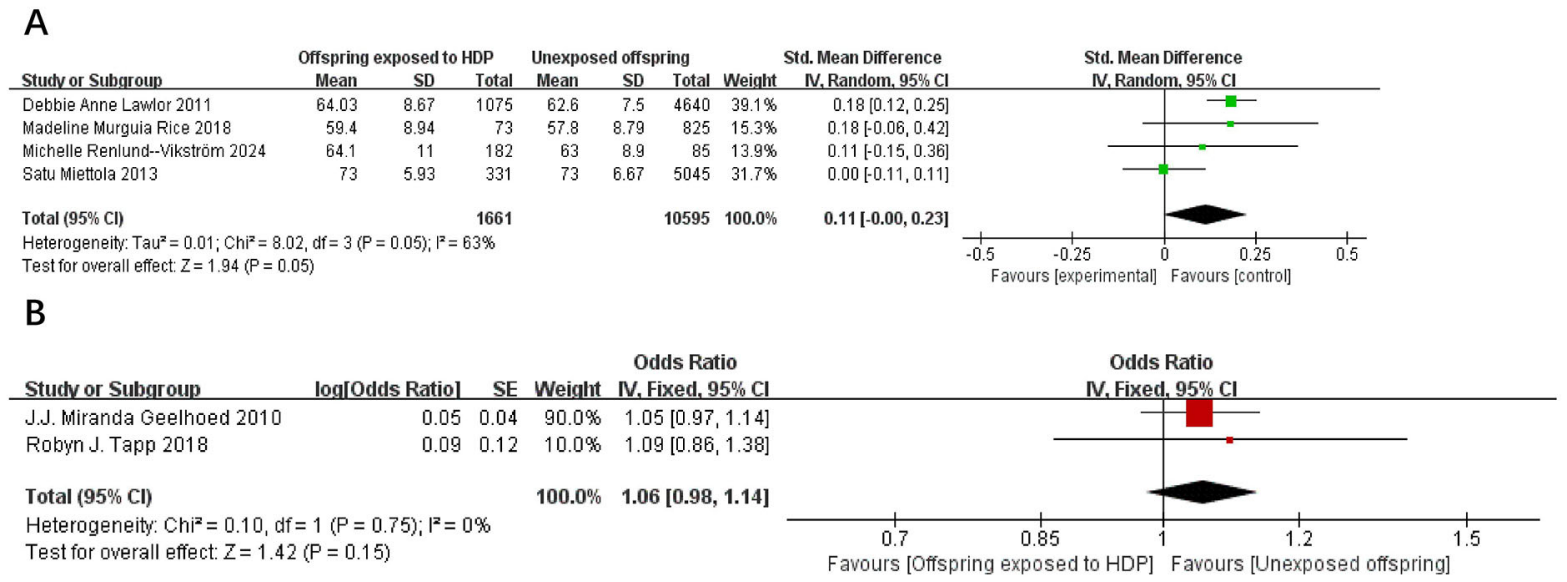
The forest plot shows the impact of HDP on offspring waist circumference, **(A)** continuous variable **(B)** categorical variable.

A total of 14,327 participants from six articles were included to evaluate the effects of HDP on offspring waist circumference. Higher offspring waist circumference is associated with greater risk. As shown in **Figures 5A, B**, in 4 studies (19, 23, 37, 39), *I*² = 63% prompted a random-effects model, yielding an SMD of 0.11 (95% CI: −0.00 to 0.23; *P* = 0.05), which was not statistically significant. In two studies (17, 20), *I*² = 0% prompted a fixed-effect model, yielding an OR of 1.06 (95% CI: 0.98–1.14; *P* = 0.15), also not statistically significant.

**Figure 6:**
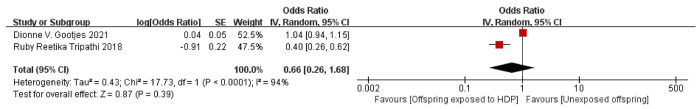
The forest plot shows the impact of HDP on offspring Fat Mass Index.

A total of 8,856 participants from 2 articles were included to assess the effects of HDP on offspring fat mass index. Higher offspring fat mass index is associated with greater risk. As shown in **Figure 6**, in these two studies (16, 30), *I*² = 94% prompted a random-effects model, yielding an OR of 0.66 (95% CI: 0.26–1.68; *P* = 0.39), which was not statistically significant.

#### 3.4.3 Results: effect of HDP on offspring lipid metabolism

**Figure 7:**
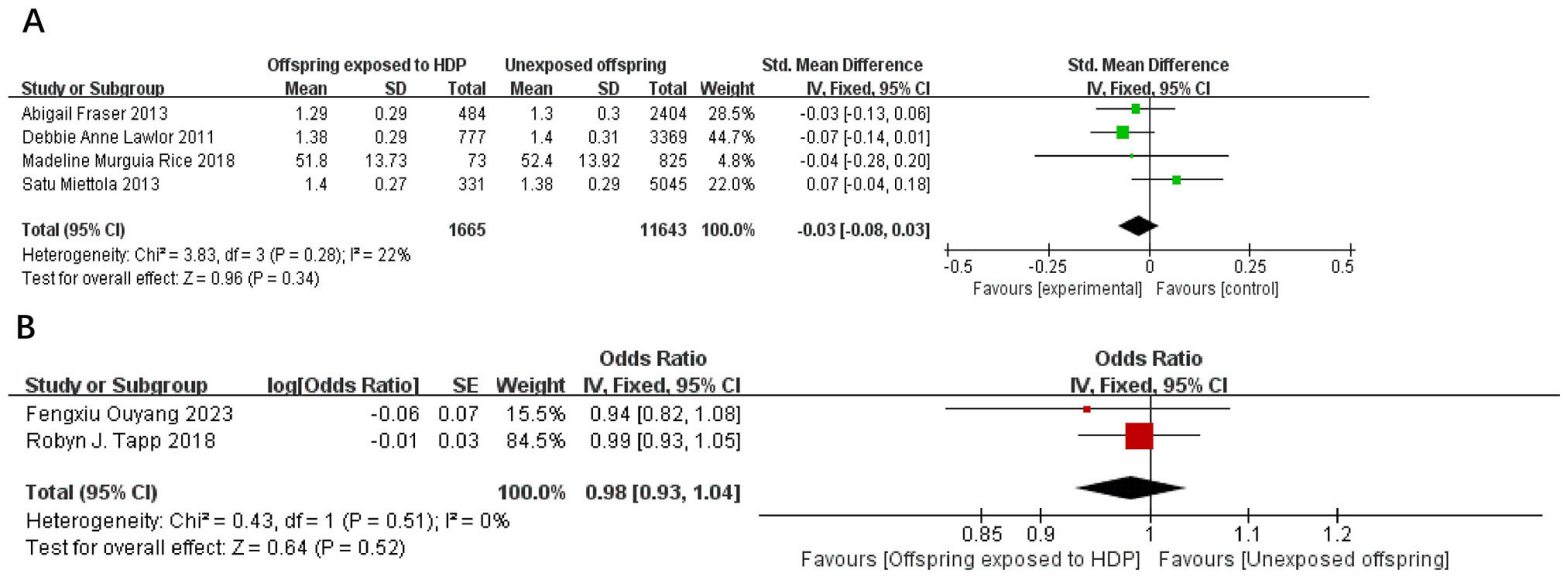
The forest plot shows the impact of HDP on offspring HDL, **(A)** continuous variable **(B)** categorical variable.

A total of 14,863 participants from 6 articles were included to assess the effects of HDP on offspring HDL. Higher offspring HDL is associated with lower risk. As shown in **Figures 7A, B**, in four studies (19, 22, 23, 37), *I*² = 22% prompted a fixed-effect model, yielding an SMD of −0.03 (95% CI: −0.08 to 0.03; *P* = 0.34; no statistical significance was found. In two studies (20, 34), *I*² = 0% prompted a fixed-effect model, yielding an OR of 0.98 (95% CI: 0.93–1.04; *P* = 0.52), also not significant.

**Figure 8:**
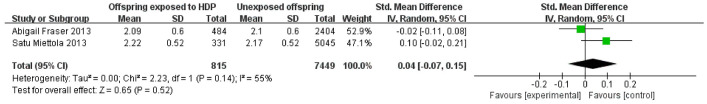
The forest plot shows the impact of HDP on offspring LDL.

**Figure 9:**
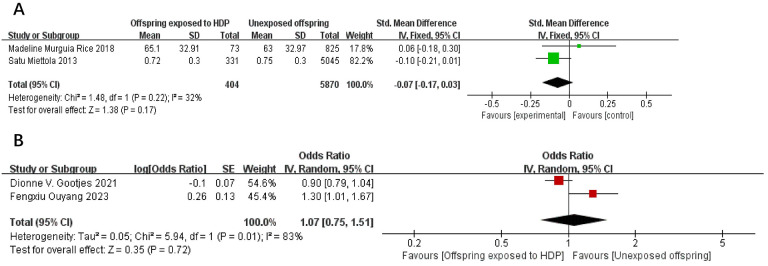
The forest plot shows the impact of HDP on offspring triglyceride, **(A)** continuous variable **(B)** categorical variable.

A total of 8,264 participants from 2 articles were included to assess the effects of HDP on offspring LDL. Higher offspring LDL is associated with greater risk. As shown in **Figure 8**, in two studies (22, 23), *I*² = 55% prompted a random-effects model, yielding an SMD of 0.04 (95% CI: −0.07 to 0.15; *P* = 0.52; no statistical significance was found. A total of 14,617 participants from four articles were included to assess the effects of HDP on offspring triglycerides. Higher offspring triglyceride levels are associated with greater risk. As shown in **Figure 9A, B**, in 2 studies (19, 23), *I*² = 32% prompted a fixed-effect model, yielding an SMD of −0.07 (95% CI: −0.17 to 0.03; *P* = 0.17),; no statistical significance was found. In 2 studies (30, 34), *I*² = 83% prompted a random-effects model, yielding an OR of 1.07 (95% CI: 0.75–1.51; *P* = 0.72), also not significant.

**Figure 10:**
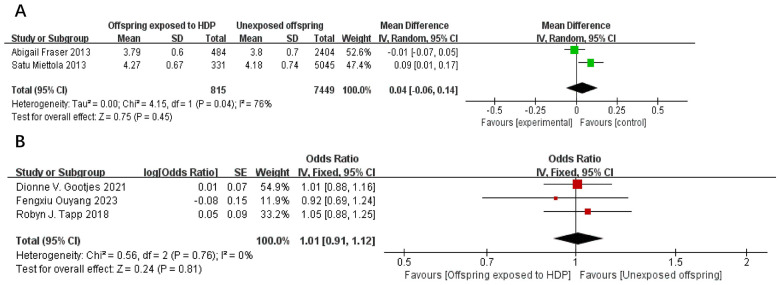
The forest plot shows the impact of HDP on offspring total cholesterol, **(A)** continuous variable **(B)** categorical variable.

A total of 17,611 participants from five articles were included to assess the effect of HDP on offspring total cholesterol. As shown in **Figures 10A, B**, in 2 studies (22, 23), *I*² = 76% prompted a random-effects model, yielding an MD of 0.04 (95% CI: −0.06 to 0.14; *P* = 0.45; no statistical significance was found. In three studies (20, 30, 34), *I*² = 0% prompted a fixed-effect model, yielding an OR of 1.01 (95% CI: 0.91–1.12; *P* = 0.81), also not significant.

#### 3.4.4 Results: impact of HDP on offspring glucose metabolism

**Figure 11:**
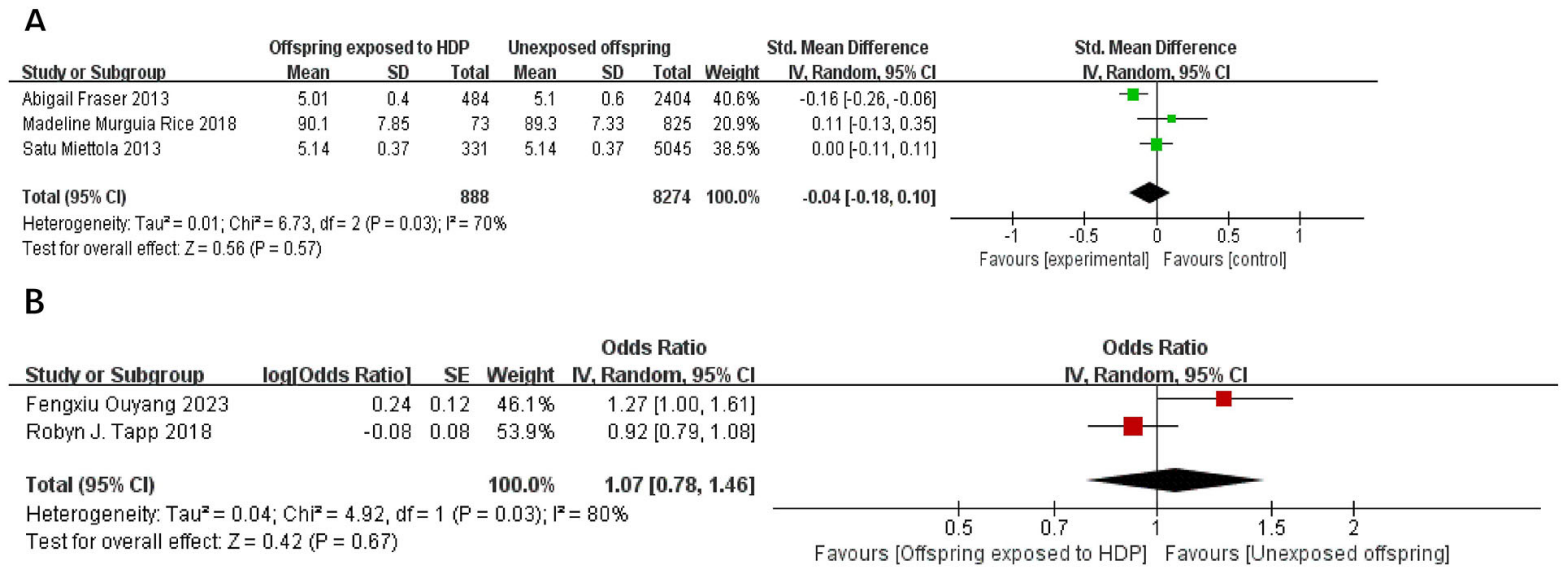
The forest plot shows the impact of HDP on offspring blood glucose, **(A)** continuous variable **(B)** categorical variable.

A total of 10,717 participants from five articles were included to assess the effects of HDP on offspring blood glucose. Higher offspring blood sugar is associated with greater risk. As shown in **Figures 11A, B**, in 3 studies (19, 22, 23), *I*² = 70% prompted a random-effects model, yielding an SMD of −0.04 (95% CI: −0.18 to 0.10; *P* = 0.57), not statistically significant. In two studies (20, 34),*I*² = 80% prompted a random-effects model, yielding an OR of 1.07 (95% CI: 0.78–1.46; *P* = 0.67), also not significant.

**Figure 12:**
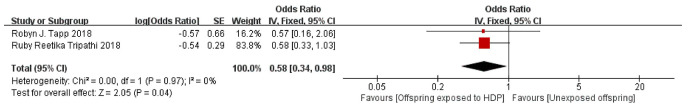
The forest plot shows the impact of HDP on offspring HOMA-IR.

A total of 2,068 participants from two articles were included to assess the effect of HDP on offspring HOMA-IR. As shown in **Figure 12**, in these two studies (16, 20), *I*² = 0% prompted a fixed-effect model, yielding an OR of 0.58 (95% CI: 0.34–0.98; *P* = 0.04), which was statistically significant.

### 3.5 Meta-regression analysis

To evaluate whether offspring age influences the association between HDP and offspring cardiometabolic indicators, a meta-regression analysis was conducted. HDP was used as the independent variable, cardiometabolic indicators (e.g., SBP, DBP, BMI) as dependent variables, and offspring age as a covariate. The regression coefficient (β) values for offspring age were small, and *P* > 0.05 (0.53–0.85), indicating no significant influence of offspring age on the association between HDP and cardiometabolic indicators. However, this finding may be affected by the relatively small number of studies. Future research should further investigate the potential impact of offspring age and consider increasing sample size to enhance statistical power. The specific results are presented in **Supplementary Table 2** (meta-regression analysis: A, continuous variables; B, categorical variables).

### 3.6 Publication bias and sensitivity analysis

**Figure 13:**
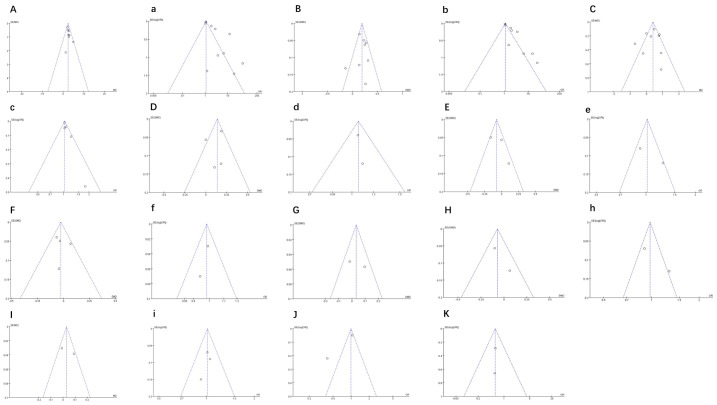
**(A, a)** Funnel plot of SBP. **(B, b)** Funnel plot of DBP. **(C, c)** Funnel plot of BMI. **(D, d)** Funnel plot of waist circumference. **(E, e)** Funnel plot of blood glucose. **(F, f)** Funnel plot of HDL. **(G)** Funnel plot of LDL. **(H, h)** Funnel plot of triglyceride. **(I, i)** Funnel plot of total cholesterol. **(J)** Funnel plot of total Fat Mass Index. **(K)** Funnel plot of HOMA-IR.

Results for 11 indicators are shown in **Figure 13**, including SBP, DBP, BMI, and waist circumference, among others. Funnel plot symmetry was evaluated to detect potential publication bias. The funnel plots were roughly symmetrical, indicating no significant publication bias.

## 4 Discussion

### 4.1 Main results

This review analyzed the cardiometabolic effects of gestational hypertension on offspring based on 23 studies, comparing them with offspring of mothers without hypertension. It included 11 outcome measures and involved 89,982 participants, representing a relatively large sample. Although follow-up periods varied, all studies included measures of HDP and cardiometabolic outcomes. The results show that offspring cardiometabolic health is affected in multiple ways by HDP: SBP, DBP, and BMI were adversely affected, while the risk of insulin resistance was lower. No significant associations were observed for blood glucose or blood lipids.

### 4.2 Interpretation (in light of other evidence)

#### 4.2.1 Effects of HDP on offspring blood pressure

This study reveals a significant link between HDP and elevated offspring blood pressure. Meta-analysis showed that offspring of hypertensive pregnant women had higher SBP (MD: 2.44; 95% CI: 2.03-2.85, *P* < 0.00001) and DBP (SMD: 0.19; 95% CI: 0.15-0.23, *P* < 0.00001). Davis et al. (13) reported a 2.39 mmHg increase in SBP in children born to preeclamptic mothers, consistent with our findings. Thoulass et al. (41) also found higher SBP in adolescence among HDP-exposed offspring. Alsnes et al. (14) showed that HDP exposure independently increased SBP by 2.2 mmHg (95% CI: 1.2–3.1) in offspring, even after adjusting for confounders. In this study, the elevated blood pressure observed in offspring exposed to HDP may be attributed to vascular endothelial dysfunction during the fetal period (42, 43). This dysfunction can alter the responsiveness of blood vessels to pressure changes, thereby increasing the risk of hypertension in these offspring. Additionally, epigenetic modifications within the renin-angiotensin system (44) may contribute to blood pressure dysregulation in HDP-exposed offspring. Glucocorticoid imbalances (23), which can affect fetal adrenal cortex function, may also play a role in blood pressure regulation. Furthermore, HDP may have lasting effects on cardiovascular development through placental dysfunction and fetal nutrition (45). These mechanisms align with the elevated blood pressure findings in our study, underscoring the importance of early intervention and long-term monitoring for offspring exposed to HDP.

#### 4.2.2 Influence of HDP on offspring obesity

Our study disclosed a significant link between HDP and offspring BMI (MD: 0.34; 95% CI: 0.05–0.64; *P* < 0.05). However, there were no significant differences noted in waist circumference or fat mass index. In a follow-up of 15,778 adolescents, Alsnes et al. (14) reported higher BMI in HDP-exposed individuals (0.66; 95% CI: 0.31–1.01). Chen et al. (35) also observed elevated BMI at age 7 in offspring of mothers with mild HDP (adjusted β: 0.03; 95% CI: 0.01–0.05). These findings suggest that mild HDP may increase the risk of offspring overweight or obesity (46). The link between HDP and offspring BMI may be mediated by epigenetic modifications that influence adipose tissue development and function (19, 47, 48) The pre-pregnancy BMI of the mother is also a significant factor, as a higher pre-pregnancy BMI is associated with an increased risk of HDP and may jointly impact offspring weight through both genetic and environmental pathways. Additionally, the intrauterine environment’s effect on adipose tissue development should not be overlooked. Yan et al. (49) highlighted a gender difference, with female offspring experiencing a significant increase in BMI (MD: 1.04; 95% CI: 0.67–1.42; *P* < 0.05), suggesting potential sex hormone regulation. This gender difference aligns with the observed increase in BMI in our study, indicating that sex hormones may play a crucial role in the impact of HDP on offspring weight.

#### 4.2.3 Effects of HDP on offspring lipid metabolism

Abnormal lipid metabolism is a significant risk factor for cardiovascular diseases in offspring. However, the relationship between HDP and offspring lipid metabolism remains controversial. Our study found no significant correlation between HDP and offspring lipid indices (HDL, LDL, triglycerides, and total cholesterol). This aligns with several large cohort studies showing no significant association between HDP and early lipid levels in offspring (20, 23, 37). However, some studies report increased dyslipidemia risk in HDP offspring in early childhood, possibly due to fetal nutritional changes, inflammation, oxidative stress, and genetic factors (22, 34). While this study did not identify a significant association between HDP and offspring lipid metabolism, several potential mechanisms have been proposed in prior research. For example, alterations in fetal nutrition, particularly during critical developmental periods, may impact lipid metabolism (22, 34). Moreover, inflammation and oxidative stress may also influence lipid metabolism in offspring exposed to HDP. Notably, Gootjes et al. found a negative association between maternal hypertension and triglyceride levels in 6-year-old girls, potentially related to sex hormone regulation of lipid metabolism (30, 50–52). In mice, preeclampsia exposure reduced lipid transporters and binding proteins in female placentas, affecting lipid metabolism (53). The “no association” conclusion must be interpreted cautiously, as dyslipidemia may take longer to manifest. Factors such as HDP severity, maternal lifestyle, and genetics may influence this relationship (30). Most studies lack long-term dynamic lipid assessments, limiting current understanding. Thus, while no direct causal link is evident, long-term follow-up studies are needed to elucidate the correlation between HDP and offspring lipid metabolism, as metabolic abnormalities may develop later in life.

#### 4.2.4 Effects of HDP on offspring glucose metabolism

Current research on how HDP impacts offspring blood glucose metabolism has yielded inconsistent results. Studies have yet to reach a consensus on this matter. In our study, offspring exposed to HDP showed a significant decrease in HOMA-IR (OR: 0.58; 95% CI: 0.34–0.98; *P* = 0.04). However, no significant correlation was observed with blood glucose levels.

Some studies report associations between maternal hypertension and abnormal offspring metabolism. For example, a Chinese study showed elevated blood glucose in 2-year-olds exposed to maternal hypertension (β = 0.24; 95% CI: 0.01–0.47) (34). Conversely, Tripathi et al. found lower insulin resistance in children (mean age 8 years) from hypertensive pregnancies (β = −0.54; 95% CI: −1.10 to 0.01) (16), while other studies found no significant associations (19, 20, 22). The impact of HDP on offspring glucose metabolism may be associated with insulin secretion and insulin sensitivity during the fetal period. In this study, the observed decrease in HOMA-IR may suggest that offspring exposed to HDP have certain advantages in terms of insulin sensitivity. This could be related to metabolic adaptation during the fetal period, such as adjustments in insulin secretion to cope with changes in the intrauterine environment. However, this advantage may not persist into adulthood, as other studies have shown inconsistent results for glucose metabolism in offspring at different ages. These inconsistencies may arise from differences in subjects’ ages, metabolic indicators, methodological approaches, and the heterogeneity of gestational hypertension. Future large-scale prospective trials with long-term follow-up are needed to clarify the long-term impact on offspring glucose metabolism and to adjust for confounding factors using a unified standard.

### 4.3 Strengths and limitations

The strengths of this study include its large sample size (n = 89,982) and its comprehensive evaluation of 11 cardiometabolic indicators across diverse populations. The findings provide a scientific basis for early intervention in HDP-exposed offspring and emphasize the importance of perinatal care in preventing cardiovascular diseases. However, limitations include the natural occurrence of HDP, long follow-up durations, potential data loss due to population mobility, and the complexity of gene–environment interactions. Additionally, diagnostic criteria for HDP are not standardized, and the prevalence of HDP varies geographically, which may affect the study’s generalizability.

## 5 Conclusion

This study systematically analyzed the impact of HDP on offspring cardiometabolic outcomes. The comprehensive nature of the review and meta-analysis provided a robust foundation for the findings. The results demonstrated that HDP is significantly associated with offspring cardiovascular and metabolic health. These effects are mainly reflected in increased systolic and diastolic blood pressure and higher body mass index, as well as a reduced risk of insulin resistance. However, no significant associations were found with other metabolic markers, including blood glucose and lipids. Further research is needed to explore the underlying mechanisms and long-term effects to better provide targeted health guidance and intervention measures for affected populations.

## Data Availability

The original contributions presented in the study are included in the article/**Supplementary Material**. Further inquiries can be directed to the corresponding author.

## Supplementary material

Supplementary Table 1 The complete search strategy for all databases

Supplementary Table 2 Meta-regression analysis (A) Continuous Variables (B) Categorical Variables.
