# Supplementary material for: Association of hypertensive disorders of pregnancy with offspring cardiometabolic indicators: a systematic review and meta-analysis
Source: Front Endocrinol (Lausanne). 2025 Nov 3;16:1641563. doi: 10.3389/fendo.2025.1641563 (PMC12620240; doi:10.3389/fendo.2025.1641563)
Supplement: Supplementary Table 1 — The complete search strategy for all databases [file Table1.doc]

|  | PubMed |
| --- | --- |
| #1 | Hypertension, Pregnancy-Induced[MeSH Terms] |
| #2 | (((((((((((Hypertension, Pregnancy-Induced[Title/Abstract]) OR (Hypertension, Pregnancy Induced[Title/Abstract])) OR (Pregnancy-Induced Hypertension[Title/Abstract])) OR (Gestational Hypertension[Title/Abstract])) OR (Hypertension, Gestational[Title/Abstract])) OR (Pregnancy Induced Hypertension[Title/Abstract])) OR (Induced Hypertension, Pregnancy[Title/Abstract])) OR (Transient Hypertension, Pregnancy[Title/Abstract])) OR (Hypertension, Pregnancy Transient[Title/Abstract])) OR (Pregnancy Transient Hypertension[Title/Abstract])) OR (HDP[Title/Abstract])) OR (HDPS[Title/Abstract]) |
| #3 | #1 OR #2 |
| #4 | ((((Cardiovascular System[MeSH Terms]) OR (Cardiovascular Diseases[MeSH Terms])) OR (Cardiometabolic Risk Factors[MeSH Terms])) OR (Metabolic Syndrome[MeSH Terms])) OR (Endocrine System[MeSH Terms]) |
| #5 | ((((((((((((((((((Cardiovascular System[Title/Abstract]) OR (Cardiovascular Diseases[Title/Abstract])) OR (Cardiometabolic Risk Factors[Title/Abstract])) OR (Metabolic Syndrome[Title/Abstract])) OR (Endocrine System[Title/Abstract])) OR (Syndromes, Metabolic[Title/Abstract])) OR (cardiovascular health[Title/Abstract])) OR (Cardiovascular diseases[Title/Abstract])) OR (Disease, Cardiovascular[Title/Abstract])) OR (metabolic health[Title/Abstract])) OR (Glucose[Title/Abstract])) OR (Birthweight[Title/Abstract])) OR (BMI[Title/Abstract])) OR (Insulin[Title/Abstract])) OR (fasting insulin[Title/Abstract])) OR (Insulin Resistance[Title/Abstract])) OR (Cholesterol[Title/Abstract])) OR (Triglycerides[Title/Abstract])) OR (Blood Pressure[Title/Abstract]) |
| #6 | #4 OR #5 |
| #7 | Child[MeSH Terms] |
| #8 | (((Child[Title/Abstract]) OR (Children[Title/Abstract])) OR (Childhood[Title/Abstract])) OR (Offspring[Title/Abstract]) |
| #9 | #7 OR #8 |
| #10 | #3 AND #6 AND #9 |

EMBASE

| #1 | 'hypertension, pregnancy-induced':ab,ti OR 'hypertension, pregnancy induced':ab,ti OR 'pregnancy-induced hypertension':ab,ti OR 'gestational hypertension':ab,ti OR 'hypertension, gestational':ab,ti OR 'pregnancy induced hypertension':ab,ti OR 'induced hypertension, pregnancy':ab,ti OR 'transient hypertension, pregnancy':ab,ti OR 'hypertension, pregnancy transient':ab,ti OR 'pregnancy transient hypertension':ab,ti OR hdp:ab,ti OR hdps:ab,ti |
| --- | --- |
| #2 | 'cardiovascular system':ab,ti OR 'cardiometabolic risk factors':ab,ti OR 'metabolic syndrome':ab,ti OR 'endocrine system':ab,ti OR 'syndromes, metabolic':ab,ti OR 'cardiovascular health':ab,ti OR 'cardiovascular diseases':ab,ti OR 'disease, cardiovascular':ab,ti OR 'metabolic health':ab,ti OR glucose:ab,ti OR birthweight:ab,ti OR bmi:ab,ti OR insulin:ab,ti OR 'fasting insulin':ab,ti OR 'insulin resistance':ab,ti OR cholesterol:ab,ti OR triglycerides:ab,ti OR 'blood pressure':ab,ti |
| #3 | child:ab,ti OR children:ab,ti OR childhood:ab,ti OR offspring:ab,ti |
| #4 | #1 AND #2 AND #3 |

Web of s

| #1 | (((((((((((AB=(Hypertension,Pregnancy-Induced)) OR AB=(Hypertension,PregnancyInduced)) OR AB=(Pregnancy-Induced Hypertension)) OR AB=(GestationalHypertension)) ORAB=(Hypertension, Gestational)) OR AB=(Pregnancy Induced Hypertension)) OR AB=(InducedHypertension,Pregnancy))ORAB=(Transient Hypertension, Pregnancy)) OR AB=(Hypertension, Pregnancy Transient)) OR AB=(Pregnancy Transient Hypertension)) OR AB=(HDP)) OR AB=(HDPS) |
| --- | --- |
| #2 | ((((((((((((((((((AB=(Cardiovascular System)) OR AB=(Cardiovascular Diseases)) OR AB=(Cardiometabolic Risk Factors)) OR AB=(Metabolic Syndrome)) OR AB=(Endocrine System)) OR AB=(Syndromes, Metabolic)) OR AB=(cardiovascular health)) OR AB=(Cardiovascular diseases)) OR AB=(Disease, Cardiovascular)) OR AB=(metabolic health)) OR AB=(Glucose)) OR AB=(Birthweight)) OR AB=(BMI)) OR AB=(Insulin)) OR AB=(fasting insulin)) OR AB=(Insulin Resistance)) OR AB=(Cholesterol)) OR AB=(Triglycerides)) OR AB=(Blood Pressure) |
| #3 | (((AB=(Child)) OR AB=(Children)) OR AB=(Childhood)) OR AB=(Offspring) |
| #4 | #1 AND #2 AND #3 |

cochrane

| #1 | Hypertension, Pregnancy-Induced):ti,ab,kw OR (Hypertension, Pregnancy Induced):ti,ab,kw OR (Pregnancy-Induced Hypertension):ti,ab,kw OR (Gestational Hypertension):ti,ab,kw OR (Hypertension, Gestational):ti,ab,kw |
| --- | --- |
| #2 | (Pregnancy Induced Hypertension):ti,ab,kw OR (Induced Hypertension, Pregnancy):ti,ab,kw OR (Transient Hypertension, Pregnancy):ti,ab,kw OR (Hypertension, Pregnancy Transient):ti,ab,kw OR (Pregnancy Transient Hypertension):ti,ab,kw |
| #3 | (HDP):ti,ab,kw OR (HDPS):ti,ab,kw |
| #4 | #1 OR #2 OR #3 |
| #5 | (Cardiovascular System):ti,ab,kw OR (Cardiovascular Diseases):ti,ab,kw OR (Cardiometabolic Risk Factors):ti,ab,kw OR (Metabolic Syndrome):ti,ab,kw OR (Endocrine System):ti,ab,kw |
| #6 | (Syndromes, Metabolic):ti,ab,kw OR (cardiovascular health):ti,ab,kw OR (Cardiovascular diseases):ti,ab,kw OR (Disease, Cardiovascular):ti,ab,kw OR (metabolic health):ti,ab,kw |
| #7 | (Glucose):ti,ab,kw OR (Birthweight):ti,ab,kw OR (BMI):ti,ab,kw OR (Insulin):ti,ab,kw OR (fasting insulin):ti,ab,kw |
| #8 | (Insulin Resistance):ti,ab,kw OR (Cholesterol):ti,ab,kw OR (Triglycerides):ti,ab,kw OR (Blood Pressure):ti,ab,kw |
| #9 | #5 OR #6 OR #7 OR #8 |
| #10 | (Child):ti,ab,kw OR (Children):ti,ab,kw OR (Childhood):ti,ab,kw OR (Offspring):ti,ab,kw |
| #11 | #4 AND #9 AND #10 |
