# Supplementary material for: Association of hypertensive disorders of pregnancy with offspring cardiometabolic indicators: a systematic review and meta-analysis
Source: Front Endocrinol (Lausanne). 2025 Nov 3;16:1641563. doi: 10.3389/fendo.2025.1641563 (PMC12620240; doi:10.3389/fendo.2025.1641563)
Supplement: Supplementary Table 2 — Meta-regression analysis (A) Continuous Variables (B) Categorical Variables. [file Table2.docx]

**Supplementary Table 2**. Meta-regression analysis (A)Continuous Variables(B)Categorical Variables

(A)

| Variable | β | SE | 95%CI_Lower | 95%CI_Upper | P_value |
| --- | --- | --- | --- | --- | --- |
| SBP | 0.013 | 0.022 | -0.03 | 0.056 | 0.55 |
| DBP | -0.009 | 0.02 | -0.048 | 0.03 | 0.65 |
| BMI | 0.02 | 0.032 | -0.043 | 0.083 | 0.53 |
| Waist | -0.008 | 0.025 | -0.057 | 0.041 | 0.75 |
| Glucose | 0.011 | 0.027 | -0.042 | 0.064 | 0.68 |
| HDL | 0.007 | 0.024 | -0.04 | 0.054 | 0.77 |
| LDL | -0.011 | 0.026 | -0.062 | 0.04 | 0.67 |
| Triglycerides | 0.016 | 0.033 | -0.049 | 0.081 | 0.63 |
| Total_cholesterol | 0.005 | 0.021 | -0.036 | 0.046 | 0.81 |

| Variable | β | SE | 95%CI_Lower | 95%CI_Upper | P_value |
| --- | --- | --- | --- | --- | --- |
| SBP | 0.01 | 0.023 | -0.035 | 0.055 | 0.66 |
| DBP | -0.008 | 0.02 | -0.047 | 0.031 | 0.69 |
| BMI | 0.015 | 0.029 | -0.042 | 0.072 | 0.6 |
| Waist_circumference | -0.011 | 0.026 | -0.062 | 0.04 | 0.67 |
| Glucose | 0.012 | 0.03 | -0.047 | 0.071 | 0.69 |
| HDL | -0.009 | 0.027 | -0.062 | 0.044 | 0.74 |
| Triglyceride | 0.018 | 0.034 | -0.049 | 0.085 | 0.6 |
| Total_cholesterol | 0.004 | 0.022 | -0.039 | 0.047 | 0.85 |
| Fat_mass_index | 0.013 | 0.031 | -0.048 | 0.074 | 0.68 |
| HOMA-IR | 0.009 | 0.028 | -0.046 | 0.064 | 0.75 |

(B)
